# Oral Versus Subcutaneous Methotrexate in Immune-Mediated Inflammatory Disorders: an Update of the Current Literature

**DOI:** 10.1007/s11926-023-01116-7

**Published:** 2023-09-28

**Authors:** Eva Vermeer, Renske C. F. Hebing, Maartje M. van de Meeberg, Marry Lin, Tim G. J. de Meij, Eduard A. Struys, Gerrit Jansen, Michael T. Nurmohamed, Maja Bulatović Ćalasan, Robert de Jonge

**Affiliations:** 1 Department of Paediatric Gastroenterology and Nutrition, Emma Children’s Hospital, Amsterdam UMC, Meibergdreef 9, 1105 AZ Amsterdam, the Netherlands; 2 Amsterdam Gastroenterology Endocrinology Metabolism Research Institute, Amsterdam UMC, Amsterdam, the Netherlands; 3 Amsterdam Reproduction and Development Research Institute, Amsterdam UMC, Amsterdam, the Netherlands; 4 Department of Rheumatology and Clinical Immunology, Amsterdam UMC, Amsterdam, the Netherlands; 5 Reade, Amsterdam Rheumatology and Immunology Centre, Amsterdam, the Netherlands; 6 Department of Gastroenterology and Hepatology, Amsterdam UMC, Amsterdam, the Netherlands; 7 Department of Laboratory Medicine, Amsterdam UMC, Amsterdam, the Netherlands; 8 Department of Rheumatology and Clinical Immunology, UMC Utrecht, Utrecht, the Netherlands

**Keywords:** Methotrexate, Methotrexate polyglutamates, Rheumatoid arthritis, Juvenile idiopathic arthritis, Inflammatory bowel disease, Crohn’s disease

## Abstract

**Purpose:**

This review aims to critically evaluate the potential benefit of either oral or subcutaneous administration of methotrexate (MTX) in various immune-mediated inflammatory disorders (IMIDs) through analysis of efficacy, toxicity, pharmacokinetics and pharmacodynamics of both administration routes.

**Recent Findings:**

Recent studies comparing the efficacy of oral versus subcutaneous MTX administration in IMIDs have revealed contradicting results. Some reported higher efficacy with subcutaneous administration, while others found no significant difference. Regarding toxicity, some studies have challenged the notion that subcutaneous administration is better tolerated than oral administration, while others have supported this. Pharmacokinetic studies suggest higher plasma bioavailability and increased accumulation of MTX-polyglutamates (MTX-PGs) in red blood cells (RBCs) with subcutaneous administration during the initial treatment phase. However, after several months, similar intracellular drug levels are observed with both administration routes.

**Summary:**

There is no conclusive evidence supporting the superiority of either oral or subcutaneous MTX administration in terms of efficacy and adverse events in IMIDs. Subcutaneous administration leads to higher plasma bioavailability and initial accumulation of MTX-PGs in RBCs, but the difference seems to disappear over time. Given the variable findings, the choice of administration route may be based on shared decision-making, offering patients the option of either oral or subcutaneous administration of MTX based on individual preferences and tolerability. Further research is needed to better understand the impact of MTX-PGs in various blood cells and TDM on treatment response and adherence to MTX therapy.

## Introduction

Methotrexate (MTX) is a folate antagonist with anti-inflammatory, immunomodulatory and anti-proliferative capacities. In low doses, MTX has been established as a first-line effective, safe and inexpensive treatment in immune-mediated inflammatory diseases (IMIDs) such as rheumatoid arthritis (RA), juvenile idiopathic arthritis (JIA) and psoriatic arthritis and as a second-line treatment in inflammatory bowel disease (IBD) [1–4]. For these indications, the drug is administered either orally or subcutaneously in dosages up to 30 mg/week.

In this narrative review, we aim to evaluate recent literature over the past 4 years on the potential benefits of oral versus subcutaneous administration of MTX therapy in RA, JIA and IBD. We discuss several aspects of MTX therapy, including clinical and biochemical response, toxicity, pharmacokinetics and MTX-polyglutamate (MTX-PGs) formation in relation to efficacy.

## Clinical Efficacy: Guidelines

The European Alliance of Associations for Rheumatology (EULAR) states that MTX should be part of the first treatment strategy in both RA and JIA, either as monotherapy or as combination therapy with another disease-modifying anti-rheumatic drug (DMARD), such as sulfasalazine or hydroxychloroquine [5]. The preferred line of treatment is however MTX monotherapy, with concomitant use of a glucocorticoid in the initiation phase. The guideline does not recommend a specific route of administration of MTX and suggests rapid dose escalation to 25 mg/week, which corresponds to a dose of 0.3 mg/kg body weight for a person weighing 80 kg. Concomitant folic acid supplementation at a dose of 5–10 mg/week is highly recommended to reduce the risk of side effects [6].

The American College of Rheumatology (ACR) similarly advises MTX as a first-line DMARD for RA and JIA at a dose of minimally 15 mg/week and is often used as primary conventional synthetic DMARD in the step-up approach to biological DMARDs, such as anti-tumour necrosis factor (TNF) agents [7, 8]. The guideline states that even though there is evidence of moderate certainty that subcutaneous administration of MTX is associated with higher efficacy, oral administration is preferred. This is mainly due to the convenience and low costs of oral administration, along with comparable bioavailability at similar starting doses. In practice, most patients typically start with oral treatment, but subcutaneous MTX is often prescribed to those who tolerate oral MTX poorly [9, 10]. For JIA, the ACR conditionally recommends subcutaneous over oral administration. This recommendation was based on several efficacy studies, of which some showed superiority of subcutaneous over oral MTX administration and some showed comparable efficacy rates [11–13]. However, the quality of evidence supporting this recommendation is considered low, and it is therefore advised to rely on shared decision-making when determining the most suitable route of administration.

The European Crohn’s and Colitis Organisation (ECCO) does not recommend MTX as a first-line treatment option in adult Crohn’s disease (CD) [14]. MTX is often prescribed to patients as a second choice when they have failed to respond to thiopurines, which are the primary treatment option for mild to moderate severe CD. Moreover, MTX is often prescribed as a complement to anti-TNF agents in order to prevent the formation of anti-drug antibodies [15]. If MTX is prescribed in adult CD, it is generally prescribed subcutaneously at the induction dose of 25 mg/week, whereas the typical maintenance dose is 15 mg/week. In paediatric CD, MTX does serve as a first-line treatment option. The paediatric ECCO guideline recommends parenteral (subcutaneous or intramuscular) MTX administration at a weekly dose of 15 mg/m^2^ to a maximum of 25 mg/week [16]. In both adult and paediatric ulcerative colitis, MTX is not recommended as monotherapy but is solely used as concomitant medication to anti-TNF agents [17–19].

## Clinical Efficacy of Oral Versus Subcutaneous MTX: Recent Studies

In the past 4 years, several studies have compared the efficacy of oral versus subcutaneous administration of MTX, all of which were conducted in the RA population. These data are displayed in Table 1.

**Table 1:** Comparison of clinical efficacy of oral vs subcutaneous administration of MTX in RA: recent literature observations

	Bujor et al. (2019)	Wang et al. (2022)	Heuvelmans et al. (2021)	Vidal-Montal. (2023)
Number of patients	703	644	640	103
MTX dose	15–25 mg/week	15–25 mg/week	15–20 mg/week	15 mg/week
Disease activity score measurement	ACR20	ACR20/50/70	DAS28-CRP	DAS28-CRP
Duration of MTX therapy	6 months	6 months	3–6 months	3 months
Achievement of outcome oral MTX	63.7% of patients achieved ACR20	70.9% of patients achieved ACR20	−1.21 (DAS28-CRP)	−0.99 (DAS28-CRP)
Achievement of outcome sc MTX	78.4% of patients achieved ACR20	78.1% of patients achieved ACR20	−1.24 (DAS28-CRP)	−1.92 (DAS28-CRP)
Difference in disease activity	OR 3.02 oral vs sc (95% CI: 1.41–6.46)	OR 0.68 sc vs oral (*p* = 0.15)	0.13 difference in DAS28-CRP (95% CI: −0.14–0.40)	0.97 difference in DAS28-CRP (*p* < 0.0001)

*ACR20/50/70* improvement rate of 20%/50%/70% based on criteria of the American College of Rheumatology, *DAS28-CRP* Disease Activity Score-28 for Rheumatoid Arthritis with CRP, *OR* odds ratio, *95% CI* 95% confidence interval, *sc* subcutaneous

Bujor et al. compared the efficacy of oral with parenteral administration of MTX in RA by conducting a meta-analysis of four studies (*n* = 703), of which three compared oral and subcutaneous MTX administration, whereas one study compared oral with intramuscular administration [20]. Dosages varied between 15 and 25 mg/week. The primary endpoint was ACR20 at 6 months, which is an achievement of at least 20% improvement in the core set measures related to disease activity. All four studies reported a higher number of patients on parenteral MTX administration attaining ACR20 at 6 months compared to oral administration. Cumulatively, patients using MTX parenterally showed a 20.2% increased chance of achieving ACR20 compared to patients using MTX orally, and the summary odds ratio (OR) was 3.02 for parenteral administration of MTX versus oral administration.

Wang et al. also performed a meta-analysis accompanied by a systematic review comparing the efficacy of oral versus parenteral MTX administration in RA at doses 15 to 25 mg/week [21••]. They included six randomised controlled trials (*n* = 644), three of which compared the efficacy, tolerability and safety of oral versus subcutaneous administration of MTX. These three trials were also included in the meta-analysis of Bujor et al. [20]. The other three trials studied the bioavailability of orally and parenterally administered MTX. The primary endpoints of this meta-analysis were ACR criteria-based response rates: ACR20, ACR50 and ACR70 (20%, 50% and 70% improvement rate). Unlike Bujor et al., Wang et al. did not find significant differences in ACR20 (OR: 0.68; *p* = 0.15), ACR50 (OR: 0.75; *p* = 0.29) and ACR70 (OR: 0.75; *p* = 0.13) between both routes of administration. Due to the lack of difference in efficacy between oral and parenteral MTX in their study, along with the higher costs and burden of injections accompanying parenteral administration, Wang et al. recommend oral over parenteral administration of MTX for treatment of active RA. It is interesting that the two meta-analyses came to a different conclusion even though they analysed the same three efficacy trials. The difference could be explained by the fact that Bujor et al. analysed the achievement of ACR20 as a primary endpoint, whereas Wang et al. looked at the achievements of ACR50 and ACR70 as well. The latter two endpoints are those that physicians preferably aim for rather than the ACR20 while assessing clinical efficacy of a drug, which arguably makes the study by Wang et al. more valuable.

Another study by Heuvelmans et al. analysed the efficacy of oral versus subcutaneous administration of MTX in a retrospectively collected cohort of 640 adult RA patients of whom 259 used MTX orally and 381 subcutaneously at dosages up to 25 mg/week [22]. The Disease Activity Score-28 for Rheumatoid Arthritis with CRP (DAS28-CRP) was used as a tool to quantify disease activity. They found significant differences in DAS28-CRP at 3 to 6 months compared to baseline for both routes of administration (−1.21 points of DAS28-CRP for oral and −1.24 points in the DAS28-CRP for subcutaneous administration of MTX). However, the difference in DAS28-CRP between the groups was not significant at a *p*-value of 0.13 (95% CI: −0.14—0.40); therefore, neither route of administration was superior.

In a comparable but smaller recent retrospective cross-sectional study, Vidal-Montal et al. compared the efficacy of oral versus subcutaneous administration of MTX in the initiation phase in 103 RA patients [23]. Sixty-three patients used MTX orally, and 40 patients used MTX subcutaneously, at mean MTX dosages of 14 mg/week and 16 mg/week, respectively. Unlike Heuvelmans et al., Vidal-Montal et al. reported a significantly lower DAS28-CRP score at 3 months after treatment initiation in the subcutaneous MTX group (−1.92 ± 1.05 DAS28-CRP points) when compared to the oral group (−0.99 ± 1.35 DAS28-CRP points), indicating superior efficacy of subcutaneous MTX administration. The difference in outcome between both studies could be explained by the smaller number of patients included in the study by Vidal-Montal et al. After 6 months, DAS28-CRP remained significantly lower in patients treated with subcutaneous MTX when compared to patients using MTX orally (2.32 ± 0.98 versus 3.01 ± 1.35) After 3 months of MTX use, 52% of patients in the oral group were in remission as opposed to 85% of patients in the subcutaneous group. After 6 months, these percentages were 65% and 83%, respectively.

Looijen et al. described a new statistical analysis on data from the TApering strategies in Rheumatoid Arthritis (TARA) trial [24]. With a linear mixed model, they compared the cumulative flare rates of patients tapering oral MTX with those of patients tapering subcutaneous MTX. The tapering regimen involved gradually reducing the dosage of MTX to half at baseline, one quarter at 3 months, and discontinuing it entirely at 6 months. Starting dosages varied between 10 and 25 mg/week. Seventeen patients in this cohort tapered subcutaneous MTX, and 71 patients tapered oral MTX. After 12 months, 53% of patients in the subcutaneous group developed a flare, compared to 27% of patients in the oral group (*p* = 0.037). Therefore, it appeared that patients tapering subcutaneous MTX have an increased risk of flare compared to patients tapering oral MTX, and they should be monitored more closely during dosage reduction. An explanation they provide for the difference in flare rates after tapering MTX is the supposed higher efficacy of subcutaneous MTX when compared to oral MTX. However, in the current review, we have shown that this difference in effectivity is not fully certain. Another remarkable detail of the study by Looijen et al. is that the subcutaneous group was substantially smaller than the oral group, which could be the reason for the observed difference in flare rates. The authors explain the difference in group size by the fact that due to common clinical practice in the region of the study, patients initially start on oral MTX and switch to subcutaneous MTX when they experience gastrointestinal side effects.

No studies have been performed in IBD comparing oral and subcutaneous administration of MTX regarding efficacy.

## Toxicity

According to the abovementioned guidelines, subcutaneous administration of MTX is more preferable regarding toxicity and tolerability since it has historically been associated with fewer side effects and adverse events [5, 7, 8]. Recent studies have questioned this and found contrasting outcomes. These data are displayed in Table 2.

**Table 2:** Comparison of MTX-treatment associated adverse events in RA: observations from recent literature

	Heuvelmans et al. (2021)		Vidal-Montal et al. (2023)		Li et al. (2021)		Wang et al. (2022)	
	Oral	SC	Oral	SC	Oral	SC	Oral	SC
Adverse events	40.5	51.7	24	30			60.1	61.3
Gastrointestinal symptoms	27.0	29.4	21	18				
Nausea							22.2	20.5
Vomiting							8.6	5
Dyspepsia							14.1	10.9
Stomatitis							4.3	3.4
Transaminitis	15.1	18.6	3	8	9	11	4.7	1.7
Neutropenia	5.4	2.9			7	7		
Hair loss	4.3	3.9						
Skin symptoms	2.3	4.7						
Mucositis	2.3	4.5						
Headache	1.9	4.5						
Fatigue	1.2	3.9	10	18				

Values in percentages (%)

*MTX* methotrexate, *RA* rheumatoid arthritis, *SC* subcutaneous

In a retrospective cohort study in 640 adult RA patients using MTX, Heuvelmans et al. compared the occurrence of side effects in oral versus subcutaneous MTX administration [22]. They found that the number of patients that reported adverse events was lower in the oral group than in the subcutaneous group. The occurrence rates of adverse events for these groups were 41% and 52%, respectively. Most common side effects, some significant, others non-significant, were gastrointestinal symptoms (27% in oral group, 29% in subcutaneous group, *p* = 0.51), hair loss (4.3% versus 3.9%, *p* = 0.81), skin symptoms (2.3% versus 4.7%, *p* = 0.12), mucositis (2.3% versus 4.5%, *p* = 0.15), headache (1.9% versus 4.5%, *p* = 0.08), fatigue (1.2% versus 3.9%, *p* = 0.04) and epistaxis (0.4% versus 2.9%, *p* = 0.02). In their recent retrospective cross-sectional study in RA, Vidal-Montal et al. reported no significant difference in the occurrence of side effects between oral administration of MTX and subcutaneous administration in the first 3 months of treatment (24% in oral group, 30% in subcutaneous group, *p* = 0.50) [23]. Similar to the findings of Heuvelmans et al., gastrointestinal side effects were most common (21% in oral group, 18% in subcutaneous group, *p* = 0.71), followed by liver enzyme elevation (3% versus 8%, *p* = 0.25) and asthenia (10% versus 18%, *p* = 0.24).

Li et al. conducted a large retrospective cohort study, assessing the safety of different DMARDs in RA by looking into the prevalence rates of transaminitis and neutropenia [25]. In this cohort, 3042 patients were treated with MTX monotherapy, 2093 of whom used MTX orally and 949 subcutaneously. They did not find a significant difference in adverse events between the two groups. Similarly, Wang et al. also did not find significant differences in odds ratios between oral and parenteral MTX for any adverse events in their meta-analysis in adult RA [21••]. In the MeMo trial, Hebing et al. also did not observe a correlation between adverse events and route of administration in adult RA patients [26•].

The most common side effects of MTX in JIA include cytopenia, elevated transaminases, oral ulcers, nausea and abdominal pain. Gastrointestinal intolerance is highly prevalent in JIA patients on MTX, affecting more than 50% of patients [27]. Interestingly, JIA patients on MTX experience gastrointestinal side effects not only upon receiving MTX but also before taking MTX (anticipatory symptoms) or when thinking of MTX (associative complaints), alongside behavioural complaints such as crying, restlessness and refusal of MTX, termed MTX intolerance [27]. Hügle et al. describe the incidence of MTX toxicity and intolerance in JIA in a narrative review [28]. They discussed several JIA studies comparing intolerance of orally administered MTX versus subcutaneously administered MTX and find contradicting outcomes. Some studies showed more gastrointestinal side effects with subcutaneous administration of MTX, while other studies showed no significant difference in the occurrence of gastrointestinal side effects between the routes of administration [12, 13, 27, 29, 30].

These outcomes are intriguing, since the switch from oral to subcutaneous administration of MTX is often practised and recommended in case of gastrointestinal side effects on orally administered MTX [31–33]. In line with the abovementioned recent studies, an older study by Bulatović Ćalasan et al. found an even higher prevalence of MTX intolerance in RA and psoriatic arthritis patients on subcutaneously administered MTX (20.6%) compared to orally administered MTX (6.2%) [30]. Furthermore, in JIA, more subcutaneous (67.5%) than oral (44.5%) users experienced MTX intolerance, which was also more severe [27].

## Pharmacokinetics: Bioavailability and MTX-PGs

**Fig. 1:**
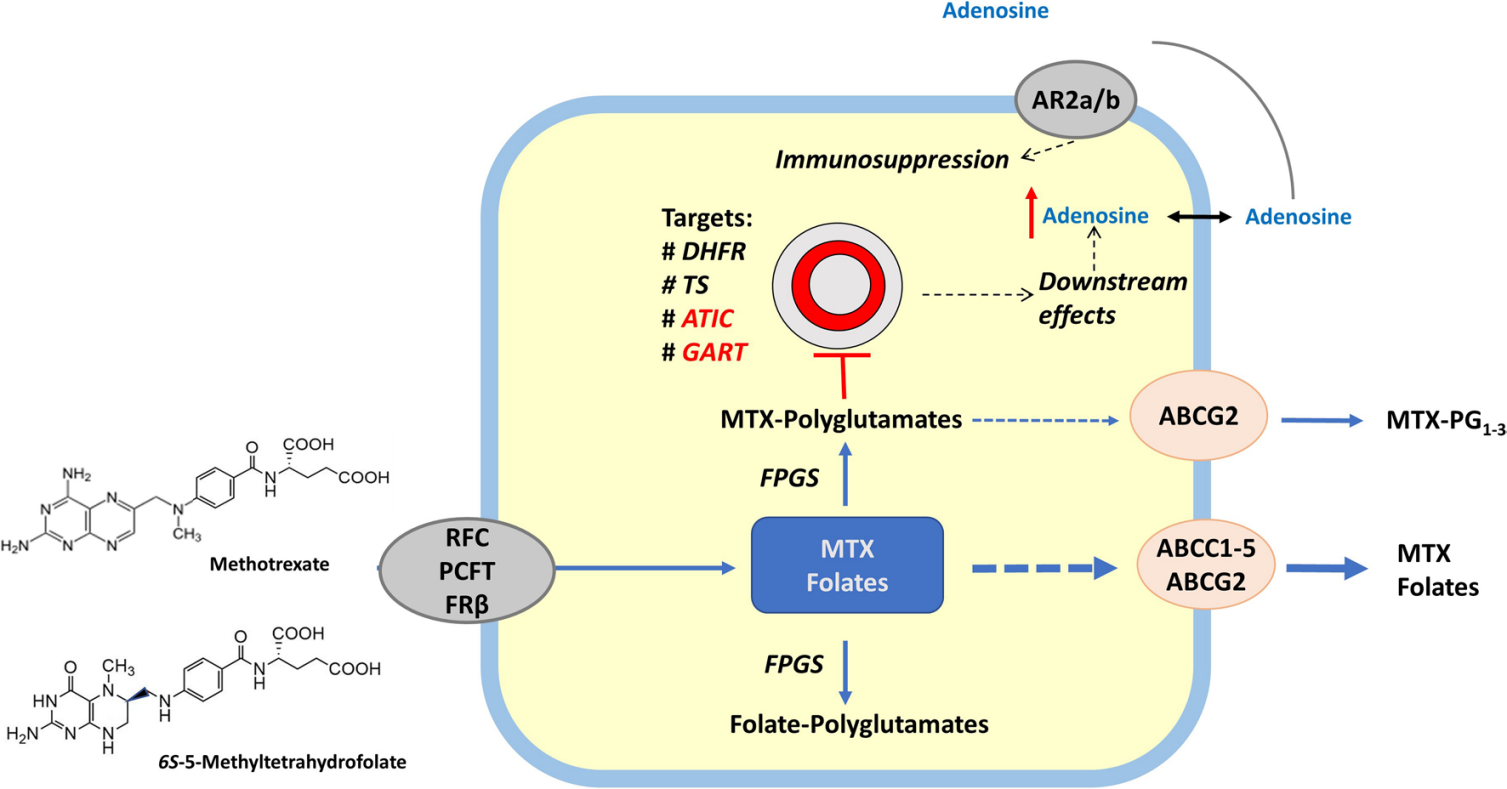
MTX cellular pharmacology and mechanism of action. Methotrexate (MTX), as well as the main circulating plasma folate 5-methyltetrahydrofolate, is taken up by (immune) target cells via 3 possible transport routes: the reduced folate carrier (RFC), proton-coupled folate transporter (PCFT) and folate receptor β (FRβ). Next, MTX is converted into MTX-polyglutamates (MTX-PGs) by the action of the enzyme folylpolyglutamate synthetase (FPGS), which are no longer substrates for ATP-binding cassette (ABCC1-5 and ABCG2) drug efflux transporters, thereby promoting their intracellular retention. MTX-PGs inhibit enzymes dihydrofolate reductase (DHFR) and thymidylate synthase (TS), but particularly, inhibition of the purine de novo enzymes GART (glycinamide ribonucleotide formyltransferase) and ATIC (5-aminiimidazole-4-carboamide ribonucleotide formyltransferase) induces upregulation of intracellular adenosine and its non-lytic extracellular release and binding to adenosine receptor 2a/b provoking an anti-inflammatory response

MTX is a folate antagonist. Following cellular uptake in immune target cells by specific folate transporters, MTX is converted to so called MTX-polyglutamates (MTX-PGs) by adding one or more glutamate residues (up to six) to the MTX. This process is catalysed by the enzyme folylpolyglutamate synthetase (FPGS) [34, 35]. MTX polyglutamylation serves two purposes; firstly, it promotes intracellular retention because of preventing drug efflux by adenosine triphosphate (ATP)-dependent exporters, and secondly, MTX-PGs potently inhibit key enzymes in the folate, pyrimidine and purine synthesis de novo pathways, the latter being involved in conferring its anti-inflammatory effect. Particularly, inhibition of the enzyme ATIC leads to intracellular accumulation of adenosine, which is a potent anti-inflammatory agent following its extracellular release, interaction with adenosine receptors and transducing immunosuppressive effects (Figure 1) [1, 36, 37]. Altogether, polyglutamylation of MTX is crucial for its intracellular retention and pharmacologic activity.

Plasma bioavailability of orally administered MTX has been known to be more variable between patients and lower than subcutaneously administered MTX [38, 39]. Oral administration of MTX has a dose-dependent plasma bioavailability of approximately 70%, reaching a plateau at 15 mg/week [38, 39]. Subcutaneous administration of MTX reaches a higher plasma availability and reaches its maximum concentration faster when compared to oral administration of MTX. Lucas et al. found that subcutaneously administered MTX provides increased and predictable plasma bioavailability [38]. In their systematic review and meta-analysis, Wang et al. also described the plasma bioavailability of MTX in RA [21••]. Their analysis supported subcutaneous MTX administration over oral administration, as subcutaneous administration of MTX resulted in slightly but nevertheless significantly higher area under the (time to plasma concentration) curves and a non-significant shorter time-to-peak plasma concentration. Plasma MTX however is eliminated within 24 h upon administration, thus begging the question what the differences are in MTX-PG concentration between oral and subcutaneous MTX.

Subcutaneous MTX administration has shown to lead to an increased accumulation of MTX-PGs in red blood cells (RBCs) in the initial treatment phase versus oral MTX administration. Hebing et al. conducted a clinical prospective cohort study in patients with RA [26•]. They randomised 43 DMARD-naive patients either to oral or subcutaneous MTX administration and measured MTX-PG concentrations in RBCs at different time points (1 to 6 months) after treatment initiation. Significantly higher MTX-PG levels were measured in RBCs in the first 2 to 3 months in the subcutaneous group compared to the oral group. However, after 3 months, no differences in RBC MTX levels were observed between the oral and subcutaneous group. Additionally, the results of this study suggest that higher concentrations of longer-chain MTX-PG (i.e. MTX-PG_3–5_) are associated with lower disease activity scores in RA, though this study was not powered to assess efficacy. In their prospective study, Hebing et al. did not find a significant difference in MTX-PG levels in peripheral blood mononuclear cells (PBMCs) in the first 3 months of MTX therapy between both route of administration groups. However, they did find that PBMCs harboured ten- to twentyfold higher MTX-PG_total_ concentrations than RBCs at all analysed time points. This could be explained by the fact that PBMCs are nucleated cells, in contrast to RBCs, which harbour a strict regulation of active folate (and thus MTX) transport, polyglutamylation and metabolism to fuel biosynthetic processes [40].

Van de Meeberg et al. performed a cross-sectional pharmacokinetic study on MTX-PGs in RBCs in adult CD patients [41]. They found a significantly lower interindividual variation of RBC MTX-PGs in patients using orally administered MTX (*n* = 7) than in patients using subcutaneously administered MTX (*n* = 12), 30.9% and 50.0%, respectively. This is a rather counterintuitive finding, since the absorption of orally administered drugs is typically more complex and subject to individual-related factors than the absorption of subcutaneously administered medication. Additionally, they observed significantly higher RBC MTX-PG_4_ and MTX-PG_5_ levels in the subcutaneous group when compared to the oral group.

## Pharmacodynamics: MTX-PGs and Efficacy

Therapeutic drug monitoring (TDM) of MTX is currently not routinely applied in IMIDs partly because stable plasma levels are not reached, plasma levels quickly fall to unmeasurably low levels after administration and validated cut-off values are lacking [42]. Several studies have suggested that intracellular MTX-PG levels could serve as potent biomarkers in predicting treatment response and disease activity in several IMIDs, including RA, JIA and IBD [43–46]. This was recently confirmed in a systematic review and meta-analysis by Van de Meeberg [47]. They included fourteen studies (*n* = 1668) in RA and three studies (*n* = 228) in JIA and concluded that higher MTX-PG levels in RBCs are associated with lower disease activity in both diseases. Subgroup analyses comparing association of MTX-PG levels and disease activity in oral versus subcutaneous MTX administration could not be performed in this meta-analysis. The identification of clinical cut-off values of MTX-PGs was beyond the scope of this meta-analysis, but twelve included studies did make suggestions regarding cut-off values associated with response or remission. For instance, six studies reported potential MTX-PG_total_ cut-off values ranging between 20 and 83.3 nmol/L [44, 45, 48–51]. For one of these studies, an MTX-PG_total_ cut-off value of 74 nmol/L associated with moderate to good response based on the EULAR criteria with a sensitivity of 87% and a specificity 64% (AUC 0.71, 95% CI 0.53–0.89, *p* = 0.034) [43], while another study found that a MTX-PG_total_ cut-off value of 83.3 nmol/L could correctly discriminate patients with ≥ 1.2 DAS28-CRP improvement from those without (AUC 0.72, 95% CI 0.57–0.87, *p* = 0.02) [45]. The variability in the found cut-off values could be explained by the differences in sample sizes, MTX dosages, route of administration, timing of MTX-PG measurement or analytical methods. More recently, Van de Meeberg et al. conducted another study on MTX-PGs in RBCs and their relation to MTX drug survival, effectivity and toxicity in adult CD (*manuscript submitted*)*.* Similarly to RA and JIA, they found that also in CD, higher MTX-PG_3_ levels were associated with improved biochemical response and MTX drug survival [44, 46]. In this study, similarly to RA, the proposed threshold level of 50 nmol/L of MTX-PG_3_ was suggested for CD patients.

## Pharmacodynamics: MTX-PGs and Adherence

Non-adherence to MTX therapy is of clinical concern impacting treatment outcomes. TDM using MTX-PG levels could potentially serve to detect incompliance and therefore determine whether a patient is failing to achieve a desired response due to ineffectiveness of the drug or rather due to incompliance. However, the threshold level for non-adherence is yet to be determined, and therefore, more extensive research on this topic is paramount.

## Conclusion

There is no convincing evidence that either route of MTX administration is superior to the other regarding efficacy in RA patients. There was also no robust evidence neither for RA nor for JIA that either route of administration gives fewer adverse events. Nevertheless, we did show conflicting data between the recently conducted two meta-analyses and two retrospective trials. However, Wang et al., who demonstrated no difference between oral and subcutaneous MTX, used more relevant outcome measures of ACR50 and 70 in comparison to Bujor et al., who chose mild improvement by ACR20. In the same vein, Heuvelmans et al., who also showed no difference in efficacy between the routes of administration, presented retrospective data of a considerably larger RA cohort than Vidal-Montal et al. All in all, recent evidence does not prove superiority of either route of administration of MTX regarding both efficacy and adverse events.

The pharmacokinetic profiles and MTX-PG accumulation in RBCs differ between oral and subcutaneous administration of MTX only during the first 2 to 3 months of treatment. Subcutaneous administration of MTX may lead to more accumulation of long-chain MTX-PGs (PG_4,5_). However, these differences in MTX-PG accumulation are not observed in PBMCs while they represent the relevant effector cells in the pathophysiology of IMIDs.

Based on the existing evidence, the optimal strategy for MTX therapeutics in IMIDs may include an approach based on shared decision-making and in the future possibly TDM for efficacy and adherence assessment, offering patients the choice between oral or subcutaneous MTX administration.

## Data Availability

All data included in this study were obtained from previously published literature.
